# Antimycobacterial and antimalarial activities of endophytic fungi associated with the ancient and narrowly endemic neotropical plant *Vellozia gigantea* from Brazil

**DOI:** 10.1590/0074-02760170144

**Published:** 2017-10

**Authors:** Mariana C Ferreira, Charles L Cantrell, David E Wedge, Vívian N Gonçalves, Melissa R Jacob, Shabana Khan, Carlos A Rosa, Luiz H Rosa

**Affiliations:** 1 Universidade Federal de Minas Gerais, Departamento de Microbiologia, Belo Horizonte, MG, Brasil; 2 United States Department of Agriculture, Natural Products Utilization Research Unit, Mississippi, USA; 3 University of Mississippi, National Center for Natural Products Research, Mississippi, USA

**Keywords:** ancient plant, antimicrobial, fungi, malaria, natural products

## Abstract

**BACKGROUND:**

Endophytic fungi, present mainly in the Ascomycota and Basidiomycota phyla, are associated with different plants and represent important producers of bioactive natural products. Brazil has a rich biodiversity of plant species, including those reported as being endemic. Among the endemic Brazilian plant species, *Vellozia gigantea* (Velloziaceae) is threatened by extinction and is a promising target to recover endophytic fungi.

**OBJECTIVE:**

The present study focused on bioprospecting of bioactive compounds of the endophytic fungi associated with *V. gigantea*, an endemic, ancient, and endangered plant species that occurs only in the rupestrian grasslands of Brazil.

**METHODS:**

The capability of 285 fungal isolates to produce antimicrobial and antimalarial activities was examined. Fungi were grown at solid-state fermentation to recover their crude extracts in dichloromethane. Bioactive extracts were analysed by chromatographic fractionation and NMR and displayed compounds with antimicrobial, antimycobacterial, and antimalarial activities.

**FINDINGS:**

Five fungi produced antimicrobial and antimalarial compounds. Extracts of *Diaporthe miriciae* showed antifungal, antibacterial, and antimalarial activities; *Trichoderma effusum* displayed selective antibacterial activity against methicillin-resistant *Staphylococcus aureus* and *Mycobacterium intracellulare*; and three *Penicillium* species showed antibacterial activity. *D. miriciae* extract contained highly functionalised secondary metabolites, yielding the compound epoxycytochalasin H with high antimalarial activity against the chloroquine-resistant strain of *Plasmodium falciparum*, with an IC_50_ approximately 3.5-fold lower than that with chloroquine.

**MAIN CONCLUSION:**

Our results indicate that *V. gigantea* may represent a microhabitat repository hotspot of potential fungi producers of bioactive compounds and suggest that endophytic fungal communities might be an important biological component contributing to the fitness of the plants living in the rupestrian grassland.

Endophytic fungi are an important source of bioactive metabolites, with a wide range of different biological activities (Strobel et al. 2004, Rosa et al. 2010). According to Strobel et al. (2004), plants from unique environmental settings, endemic species, and those with unusual longevity can be interesting targets for the recovery of unique endophytic species able to produce bioactive compounds. According to Rosa et al. (2011), endophytic fungi include a high diversity of species, mainly in the Ascomycota and Basidiomycota phyla and their anamorphs, associated with different plants around the world and representing important producers of bioactive natural products.

Brazil has a rich biodiversity of plant species, including those reported as being endemic. Among the endemic plants of the rupestrian grasslands, those of Velloziaceae occur at a high frequency and contain approximately 240 predominately neotropical species and several other species (Menezes et al. 1994, Lousada et al. 2011). *Vellozia*, known locally as ‘canela-de-ema’, is the largest genus in the family and includes approximately 105 species (Menezes et al. 1994). *Vellozia gigantea* N. L. Menezes & Mello-Silva (Velloziaceae), a recently described species, is threatened by extinction (Lousada et al. 2011). According to Alves (1994), the dracenoid species of *Vellozia*, like *V. gigantea*, may represent an ancient plant that could be hundreds of thousands of years old. In the present study, we focused on exploring the tropical endophytic fungi of *V. gigantea* as a source of antimicrobial and antimalarial compounds for use as prototype molecules to treat neglected tropical diseases.

## MATERIALS AND METHODS

*Isolation of endophytic fungi* - The endophytic fungi were recovered from leaves and adventitious roots of the endemic neotropical plant *V. gigantea* from the Brazilian rupestrian grasslands (Ferreira et al. 2017). The fungi were obtained from the Culture Collection of Microorganisms and Cells of the Federal University of Minas Gerais to cultivate and produce their crude extracts.

*Fungal cultivation and preparation of extracts for biological assays* - All fungal isolates were cultivated according to protocols established by Rosa et al. (2013). In brief, a 5-mm-diameter plug of each isolate was placed on 20 mL of PDA medium at the centre of the Petri dishes (90 mm diameter) and cultured for 15 days at 25 ± 2ºC (enough time for fungi to produce secondary metabolites). These fungal cultures were lyophilised for 72 h, cut into small pieces, and transferred to 50-mL glass centrifuge tubes, to which 50 mL of dichloromethane (DCM; Fisher Scientific, USA) was then added. After 72 h at room temperature, the organic phase was filtered, and the solvent was removed under rotary evaporation at 40ºC. An aliquot of each dried extract was dissolved in dimethyl sulphoxide (Merck, USA) to prepare a 100-mg mL^-1^ stock solution, which was stored at -20ºC. Sterile PDA medium was extracted under the same procedure and used as the control in the screening tests.

*Assays for antimicrobial activity* - Susceptibility testing of the fungal extracts, fractions and compounds from purification of extracts were performed using *Candida albicans* ATCC 90028, *C. glabrata* ATCC 90030, *C. krusei* ATCC 6258, *Cryptococcus neoformans* ATCC 90113, *Aspergillus fumigatus* ATCC 204305, *Staphylococcus aureus* ATCC 29213, methicillin-resistant *S. aureus* ATCC 33591 (MRS), *Escherichia coli* ATCC 35218, *Pseudomonas aeruginosa* ATCC 27853, and *Mycobacterium intracellulare* ATCC 23068. All microorganisms were obtained from the American Type Culture Collection (Manassas, VA) and tested using versions of the CLSI (formerly NCCLS) methods (CLSI 2002a, b, 2003, 2006). A bioassay test on *M. intracellulare* was performed as previously described with modifications (Franzblau et al. 1998). Samples were serially diluted in 20% DMSO/saline and transferred in duplicate to 96-well flat bottom microplates. Microbial inocula (1-2×10^8^ bacterial cells mL^-1^) were prepared by correcting the OD_630_ of microbe suspensions in incubation broth to create final target inocula. Ciprofloxacin (ICN Biomedicals, Ohio) at 1 µg mL^-1^ for bacteria and amphotericin B (ICN Biomedicals, Ohio) at 5 µg mL^-1^ for fungi were included in each assay as positive controls. All assayed microorganisms were read at either 530 nm using the Biotek Powerwave XS plate reader (Bio-Tek Instruments, Vermont) or 544ex/590em (*M. intracellulare, A. fumigatus*) using the Polarstar Galaxy Plate Reader (BMG LabTechnologies, Germany) prior to and after incubation. Percent growth was plotted versus test concentration to determine the IC_50_.

*Assay for screening antimalarial activity and cytotoxicity* - The antimalarial activity was determined against strains of *Plasmodium falciparum* chloroquine sensitive (D6) and chloroquine resistant (W2) by measuring plasmodial LDH activity (Makler & Hinrichs 1993). A suspension of red blood cells infected with the D6 or W2 strain of *P. falciparum* (200 μL, with 2% parasitaemia and 2% haematocrit in RPMI 1640 medium supplemented with 10% human serum and 60 μg mL^-1^ Amikacin) was added to the wells of a 96-well plate containing 10 μL of serially diluted samples (fungal extracts, fractions or pure compounds). The plate was incubated at 37ºC for 72 h in a modular incubation chamber with 90% N_2_, 5% O_2_, and 5% CO_2_. Parasitic LDH activity was determined by mixing 20 μL of the incubation mixture with 100 μL of Malstat reagent and incubating at room temperature for 30 min. Twenty microlitres of a 1:1 mixture of NBT/PES (Sigma, St. Louis, MO) was added and the plate incubated in the dark for 1 h. The reaction was stopped by adding 100 μL of a 5% acetic acid solution, and the absorbance was read at 650 nm. Chloroquine at 10.33 ng mL^-1^ for D6, 137.65 ng mL^-1^ for W2; and Artemisinin at 2.87 ng mL^-1^ for D6 and 3.21 ng mL^-1^ for W2 were included as the drug controls. IC_50_ values were computed from the dose response curves of growth inhibition using XLfit 4.2.0. The *in vitro* cytotoxicity to mammalian cell samples was tested to determine the selectivity index of the antimalarial activity. The assay was performed in 96-well tissue culture-treated plates. Vero cells (monkey kidney fibroblasts) were seeded to the wells of 96-well plate at a density of 25,000 cells well^-1^ and grown for 24 h. Samples at different concentrations were added and the cells were incubated for 48 h. Cell viability was determined by the Neutral Red method at 40 µg mL^-1^ (Borenfreund et al. 1990). Absorbance was recorded at 540 nm with an enzyme-linked immune assay-type microtiter plate reader. IC_50_ values were obtained from dose response curves.

*NMR spectroscopy* - Bioactive fungal extracts, fractions and pure compounds were analysed by NMR spectroscopy on a Bruker UXNMR 500 MHz spectrometer (Billerica, MA, USA). ^1^H and ^13^C NMR spectra recorded in DMSO-d_6_ using a standard ^1^H NMR pulse program.

*Crude extract preparation for bioassay-directed purification* - Five-millimetre-diameter plugs of each fungal isolate were placed onto 20 mL of PDA medium at the centre of 350 Petri dishes (90 mm diameter) and cultured for 15 days at 25 ± 2ºC. The fungal cultures were lyophilised for 72 h, cut into small pieces, and transferred to 50-mL glass centrifuge tubes, to which 50 mL of DCM was then added. After 72 h at room temperature, the organic phase was filtered, and the solvent was removed under rotary evaporation at 40ºC. Initially, 1.149 g of *Diaporthe miriciae* UFMGCB 9720 extract was adsorbed to silica gel and applied to a silica gel chromatography column (40-63 μm, 40 × 150 mm, 60 Å) in a Biotage XP-Sil system. The column was eluted at a flow rate of 40 mL min^-1^ using hexane/EtOAc mixtures with the following gradient: 100% hexane: 0% EtOAc to 0% hexane:100% EtOAc over 3,024 mL, and finishing with a 350-mL MeOH wash. The column eluate was collected in 27-mL fractions and, based on TLC similarities performed with the solvent hexane/EtOAc, recombined into 9 fractions [(A) 1-16, 7.7 mg; (B) 17-20, 9.1 mg; (C) 21-29, 441.4 mg; (D) 30-40, 9.3 mg; (E) 41-43, 11.2 mg; (F) 44-55, 133.8 mg; (G) 56-87, 46.2 mg; (H) 87-112, 12 mg; and (I) wash column, 479.3 mg]. Fractions H and I were selected for further investigation based on their activities in the antimalarial assays against *P. falciparum*. TLC and ^1^H NMR analyses displayed the same chemical profile for fractions H and I. Fraction I was selected and adsorbed to silica gel and applied to the silica gel chromatography column. Elution of the column was performed using increasing polarity mixtures of hexane:isopropyl alcohol in a series of 4 linear steps as follows: (step 1) 100:0 to 80:20 over 2,400 mL, (step 2) 80:20 to 50:50 over 1,200 mL, (step 3) 50:50 to 0:100 over 152 mL, and (step 4) 0:100 over 396 mL. The column eluate was collected into 27-mL portions and, based on TLC similarities performed with the solvent hexane/IPA, recombined into six fractions [(A’) 1-54, 18.8 mg; (B’) 55-59, 69.5 mg; (C’) 60-70, 168.7 mg; (D’) 71-77, 15.2 mg; and (E’) 78-169, 23.5 mg]. Fractions B’ and C’ were identified as epoxycytochalasin *H* and selected for further investigation based on their activities in the antimalarial assays.

*Identification of epoxycytochalasin H* - ^13^C NMR (120 MHz in DMSO-d_6_) δ 174.27 (C-1), 170.04 (C-21Ac), 138.67 (C-20), 137.11 (C-1ˊ), 134.13 (C-14), 129.58 (C-2ˊ and 6ˊ), 128.49 (C-13), 128.36 (C-3ˊ and 5ˊ), 126.52 (C-4ˊ), 124.49 (C-19), 75.39 (C-21), 72.22 (C-18), 62.34 (C-7), 56.74 (C-6), 53.68 (C-17), 53.52 (C-9), 53.23 (C-3), 48.52 (C-4), 44.80 (C-10), 44.67 (C-8), 42.56 (C-15), 35.89 (C-5), 30.45 (C-23), 27.58 (C-16), 26.05 (C-22), 20.44 (C-21Ac), 19.21 (C-12), 11.95 (C-11). ^13^C NMR data (Supplementary data, Figure) for fraction B’ indicated complete agreement with a previous report (Izawa et al. 1989) providing structural confirmation as epoxycytochalasin H.

## RESULTS

Among all fungal extracts screened, five displayed at least one biological activity against the different targets. Among them, the extracts of *D. miriciae* UFMGCB 9720 showed antifungal and antibacterial activities, with MIC ranging from 9. 98 to 148.79 µg mL^-1^, and antimalarial activities with 94% inhibition (Table I). *Trichoderma effusum* displayed selective antibacterial activity against methicillin-resistant *S. aureus* and *M. intracellulare*. Three *Penicillium* species (*P. herquei, P. adametzii*, and *P. quebecense*) showed antibacterial activity.

**TABLE I:** Antifungal, antibacterial and antimalarial activities of endophytic fungal extracts associated with the plant *Vellozia gigantea*

Fungal species	UFMGCB^*a*^	Yeasts^*b*^				Filamentous fungi^*b*^	Bacteria				Actinobacteria^*b*^	Malaria^*c*^
				
		CA	CG	CK	CN	AF	SA	SA MRS	EC	PA	MI	PC(D6)^*d*^
*Diaporthe miriciae*	9720	148.79	11.40	40.83	> 200	> 200	> 200	65.80	> 200	> 200	> 200	94
*Trichoderma effusum*	9736	> 200	> 200	> 200	> 200	> 200	> 200	64.60	> 200	> 200	31.98	24
*Penicillium herquei*	9829	> 200	> 200	> 200	> 200	> 200	24.40	9.98	> 200	> 200	> 200	17
*P. adametzii*	9894	> 200	> 200	> 200	> 200	> 200	> 200	23.24	> 200	> 200	> 200	36
*P. quebecense*	9928	10.81	18.81	14.89	> 200	> 200	> 200	>200	> 200	> 200	> 200	39
Control drugs	Amphotericin B	0.28	0.29	0.63	0.31	1.43	-	-	-	-	-	-
	Ciprofloxacin	-	-	-	-	-	0.13	0.11	0.01	0.09	0.40	-
	Chloroquine	-	-	-	-	-	-	-	-	-	-	90
	Artemisinin	-	-	-	-	-	-	-	-	-	-	92

a: culture of microorganisms and cells of the Universidade Federal of Minas Gerais; b: concentration which the extract displayed activity against yeasts, filamentous fungi, bacteria and actinobacteria; c: against *Plasmodium falciparum* the extract was assayed at 15.866 µg mL^-1^ and the results showed in (d) percentage of inhibition. CA: *Candida albicans*; CG: *C. glabrata*; CK: *C. krusei*; CN: *Cryptococcus neformans*; AF: *Aspergillus fumigatus*; SA: *Staphylococcus aureus*; SA MRS: *S. aureus* methicillin resistant; EC: *Escherichia coli*; PA: *Pseudomonoas aeruginosa*; MI: *Mycobacterium intracellulare*; PC (D6) *P. falciparum* chloroquine sensitive.

All bioactive extracts were examined using ^1^H NMR analysis for the presence of secondary metabolites with interesting chemical shifts. The extracts of *T. effusum, P. herquei, P. adametzii*, and *Diaporthe* sp. showed only the presence of fatty acids and, for this reason, they were not subjected only to bioassay-directed purification. In contrast, the *D. miriciae* UFMGCB 9720 extract showed the presence of highly functionalised secondary metabolites because of the presence of protons in the aromatic and olefinic regions. Detection of such compounds is clear from NMR chemical shifts, indicative of olefinic protons, aromatic protons, oxygenated methylene protons, and olefinic methyl protons. Therefore, the extract of *D. miriciae* was fractioned, yielding 238.2 mg of the compound epoxycytochalasin H (Figure), which displayed high antimalarial activity against both chloroquine-sensitive and chloroquine-resistant strains of *P. falciparum*, with IC_50_ values of 52 and 39 ng mL^-1^, respectively, without any cytotoxicity towards mammalian kidney (Vero) cells (Table II). The IC_50_ of epoxycytochalasin H in the chloroquine-resistant strain was approximately 3.5-fold lower than that of the control drug chloroquine (Table II).

**Figure:**
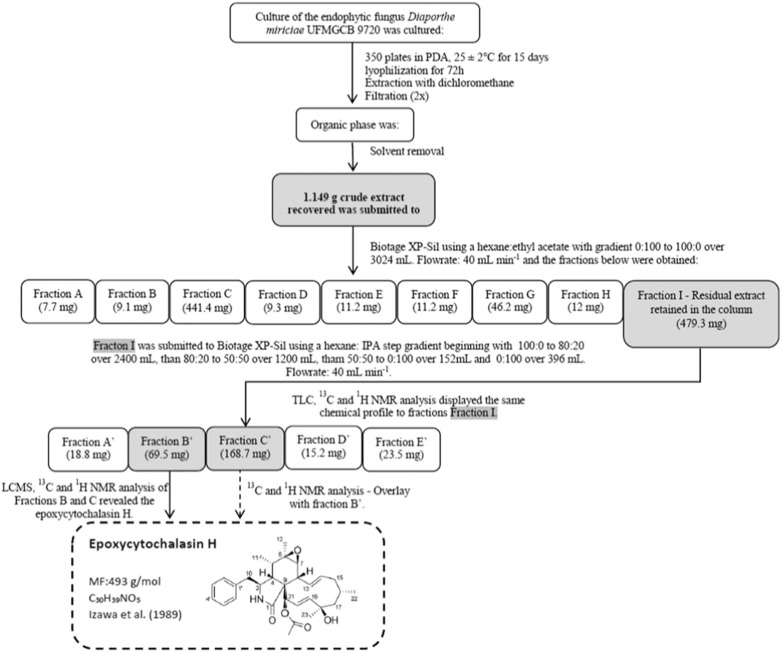
Flowcharts illustrating the processes of chemical isolation of the compound epoxycytochalasin H obtained from the endophytic fungus *Diaporthe miriciae* UFMGCB 9720.

**TABLE II:** Antimalarial activity of the compound epoxycytochalasin H isolated from the endophytic fungus *Diaporthe miriciae* UFMGCB 9720

Compound	*Plasmodium falciparum*				

	D6^*a*^ - IC_50_	D6^*a*^ - SI	W2^*b*^ - IC_50_	W2^*b*^ - SI	VERO^*c*^ IC_50_
Epoxycytochalasin H	51.70	> 92.10	39.40	> 120.70	> 4760
Chloroquine^d^	10.33	>23	137.65	> 1.70	> 238
Artemisinin^d^	2.87	>83	3.21	> 74	> 238

a: *Plasmodium falciparum* chloroquine sensitive (D6); b: *P. falciparum* chloroquine resistant (W2); c: Cell VERO; d: control drug; IC_50_: inhibitory concentration of 50%; SI: selectivity index. Values calculated at ng mL^-1^.

## DISCUSSION

*Diaporthe* was the most abundant genus of endophytes recovered associated with *V. gigantea*. Additionally, the extract of *D. miriciae* UFMGCB 9720 showed antifungal, antibacterial, and antimalarial activities. The genera *Diaporthe* and *Phomopsis* form an anamorph/teleomorph complex, which is a known producer of different bioactive compounds (Agusta et al. 2006, Carvalho et al. 2012, Silva-Hughes et al. 2015). According to Thompson et al. (2015), *Diaporthe* species are known to be saprobic and pathogenic fungi but have also been reported as endophytes in a wide range of host plants. *D. miriciae* is a new species recently described by Thompson et al. (2015); it was obtained from the plant species *Glycine max, Helianthus annuus*, and *Vigna radiata* in Australia. According to Thompson et al. (2015), *D. miriciae* forms clusters of *Diaporthe sojae*, a pathogen of *Glycine* species, suggesting that it may also be a pathogen.

The endophyte *D. miriciae* UFMGCB 9720 produced epoxycytochalasin H, which displayed high antimalarial activity against chloroquine-resistant *P. falciparum*. The cytochalasins are structurally complex secondary metabolites with more than 80 molecules described and have been isolated from fungi of the genera *Aspergillus, Diaporthe*/*Phomopsis, Penicillium, Zygosporium, Chaetomium, Phoma, Xylaria, Hypoxylon*, and *Rhinocladiella* (Dagne et al. 1994, Wagenaar et al. 2000, Zhang et al. 2012, 2014). Cytochalasins are a class of metabolites produced by fungi with antimicrobial, antitumour, anti-HIV, and herbicidal activities (Cimmino et al. 2008, Lin et al. 2009, Xu et al. 2009). Epoxycytochalasin H is produced by the soybean pathogen *Phomopsis sojae* (Cole et al. 1982) and by a *Phoma* sp. obtained from a soil sample (Kakeya et al. 1997). To the best of our knowledge, a unique activity of epoxycytochalasin H is its ability to function as a cell-cycle inhibitor in mammals (Kakeya et al. 1997).

In conclusion, our results indicate that *V. gigantea* shelters cryptic fungal species able to produce bioactive compounds in its tissues. According to Compant et al. (2016), the interaction with endophytes may be beneficial to the plant’s fitness because recent studies of plant-soil-microbe interactions revealed the potential of some endophytic fungi as promising sources of secondary metabolites for use in agriculture and medicine. Thus, epoxicitocalasin H, with activity against *P. falciparum* reported for the first time, can be used as a prototype molecule to study antimalarial substances. Our results suggest that endophytic fungal communities may be an important biological component contributing to the fitness of the plants living in the rupestrian grassland and that those plants may represent a microhabitat repository hotspot of potential fungi producers of bioactive compounds.
