# Supplementary material for: Antimycobacterial and antimalarial activities of endophytic fungi associated with the ancient and narrowly endemic neotropical plant Vellozia gigantea from Brazil
Source: Mem Inst Oswaldo Cruz. 2017 Oct;112(10):692–7. doi: 10.1590/0074-02760170144 (PMC5607518; doi:10.1590/0074-02760170144)
Supplement: Supplementary file 1 [file 0074-0276-mioc-112-10-0692-suppl01.pdf]

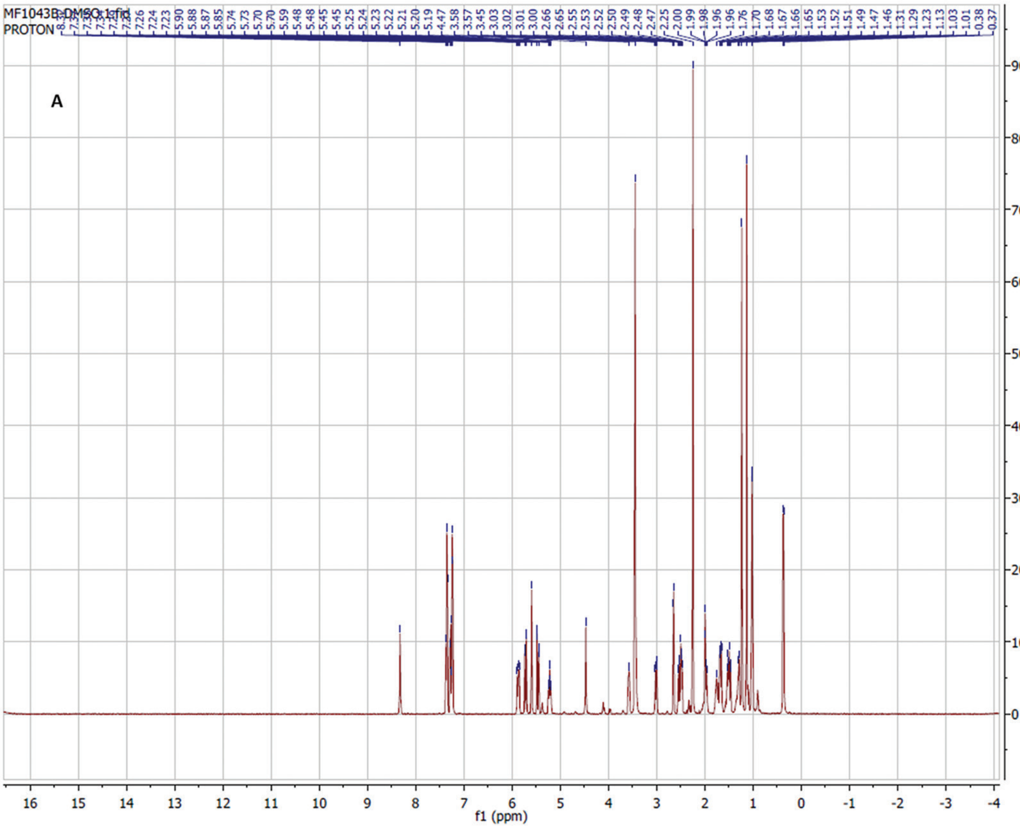
MF1043B-DMSO.1.fid
PROTON
A
f1 (ppm)

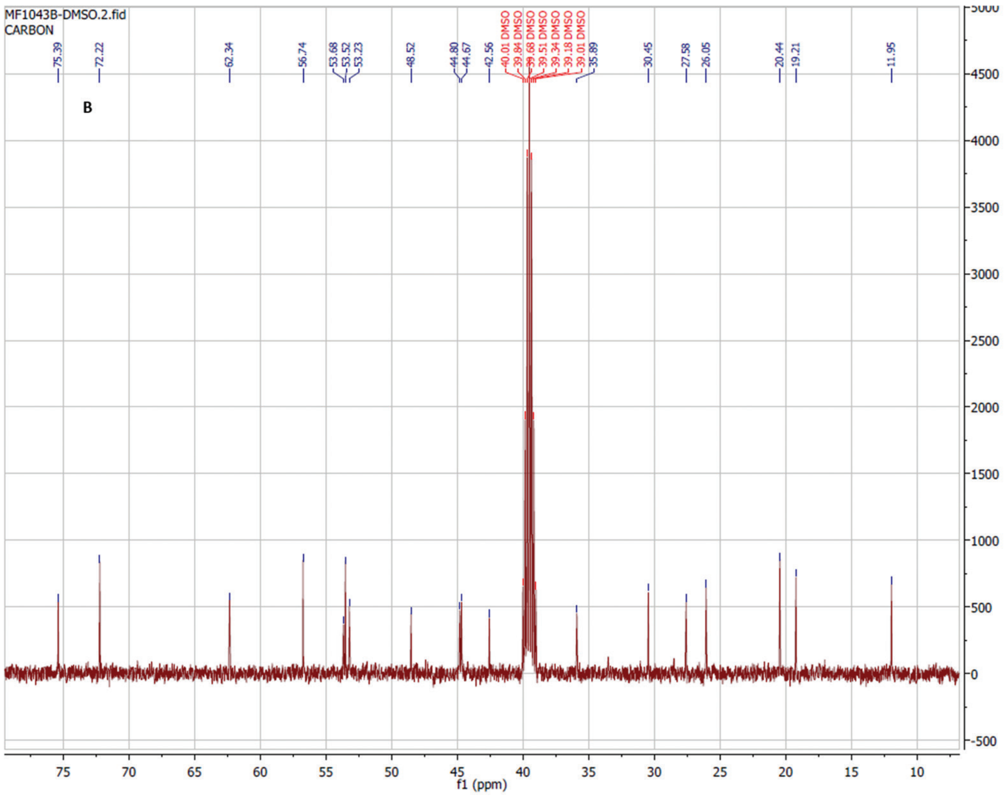
MF1043B-DMSO.2.fid
CARBON
B
75.39
72.22
62.34
56.74
53.68
53.52
53.23
48.52
44.80
44.67
42.56
40.01 DMSO
39.84 DMSO
39.68 DMSO
39.51 DMSO
39.34 DMSO
39.18 DMSO
39.01 DMSO
35.89
30.45
27.58
26.05
20.44
19.21
11.95
f1 (ppm)

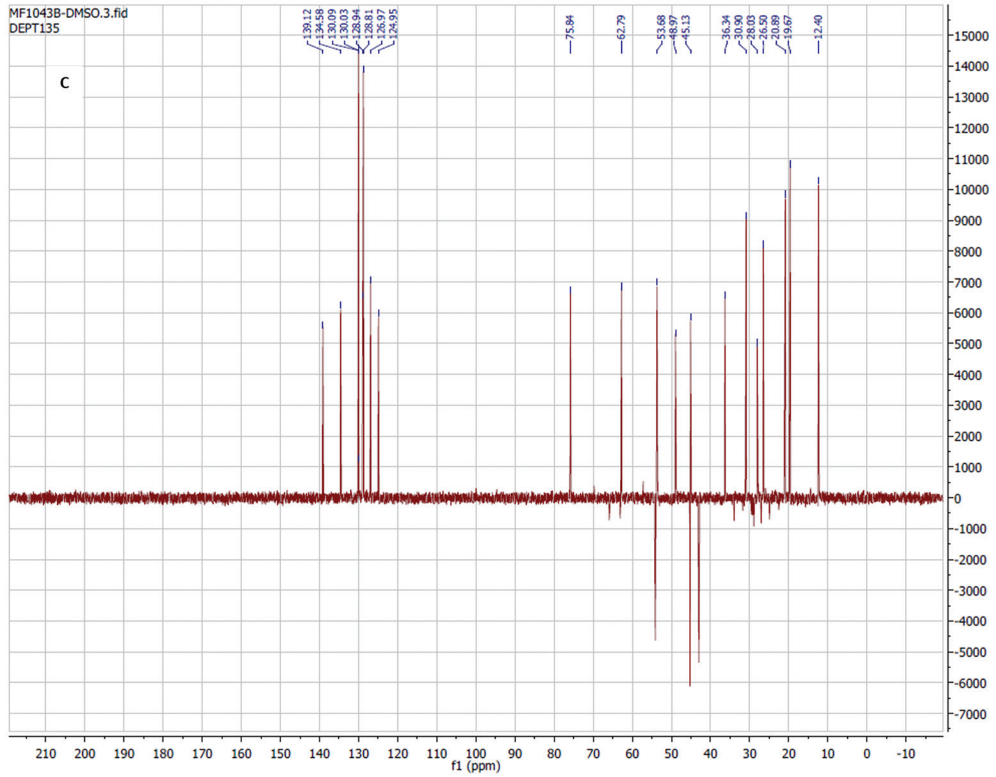
MF1043B-DMSO.3.fid
DEPT135
C
139.12
134.58
130.09
130.03
128.94
128.81
126.97
124.95
75.84
62.79
53.68
48.97
45.13
36.34
30.90
28.03
26.50
20.89
19.67
12.40
f1 (ppm)

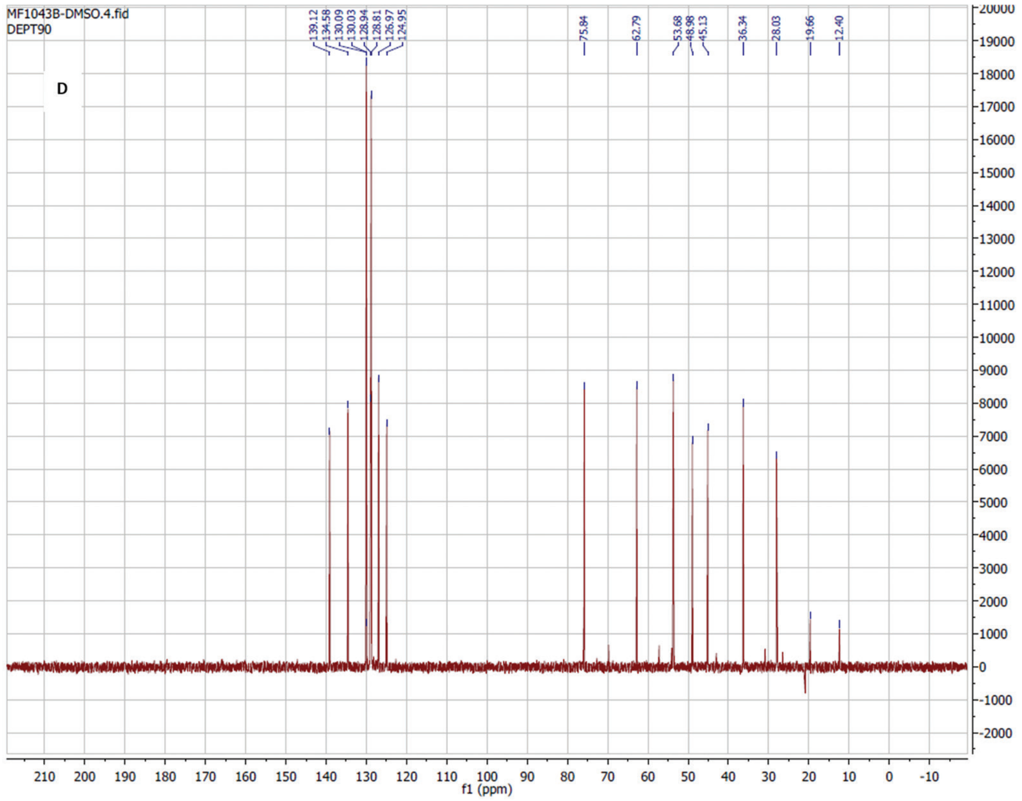
MF1043B-DMSO.4.fid
DEPT90
D
139.12
134.58
130.09
130.03
128.94
128.81
126.97
124.95
75.84
62.79
53.68
48.98
45.13
36.34
28.03
19.66
12.40
f1 (ppm)

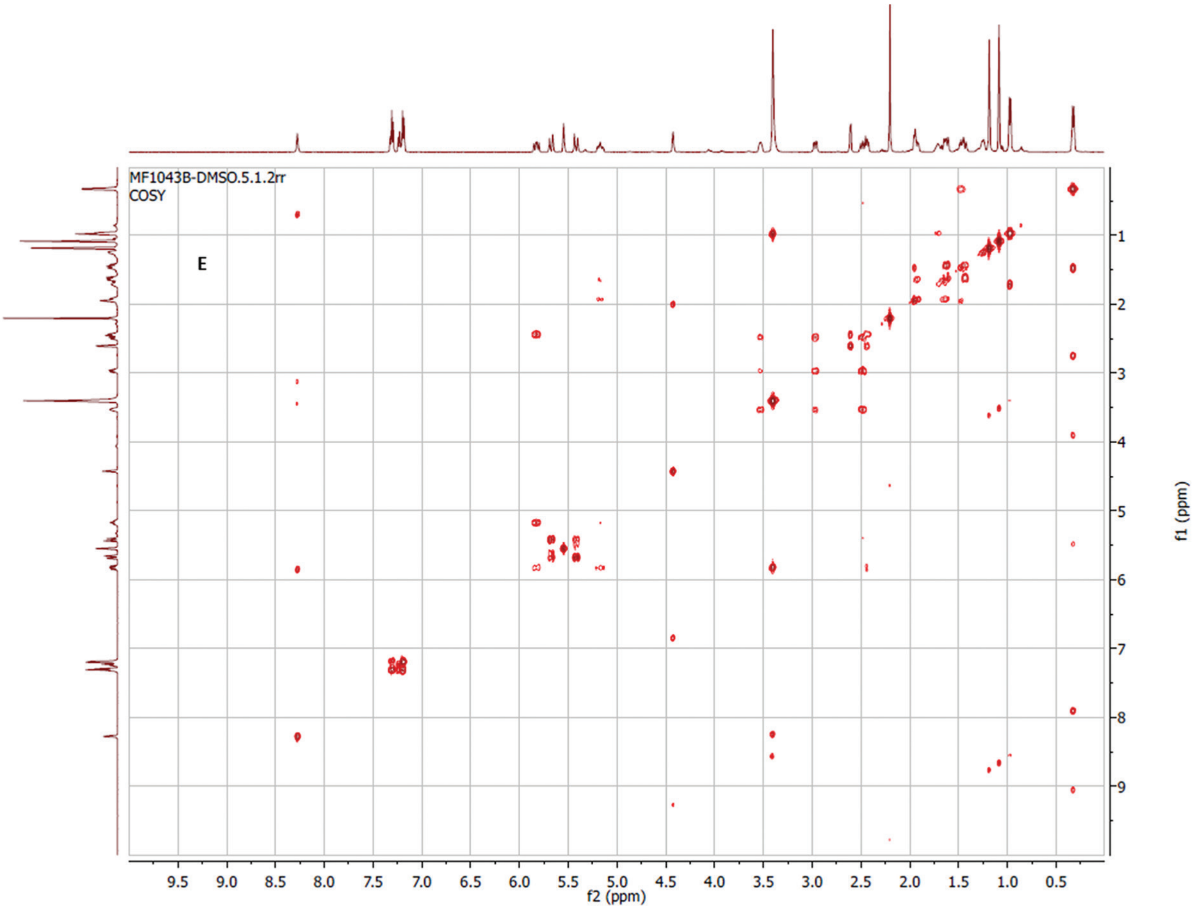

Nuclear Magnetic Resonance spectra of the epoxycytochalasin H. (A) = proton; (B) = carbon; (C) = DEPT135; (D) = DEPT 90; (E) = COSY.
